# Supplementary material for: Revisiting the functional significance of binocular cues for perceiving motion-in-depth
Source: Nat Commun. 2018 Aug 29;9:3511. doi: 10.1038/s41467-018-05918-7 (PMC6115357; doi:10.1038/s41467-018-05918-7)
Supplement: Supplementary file 1 — Supplementary Information [file 41467_2018_5918_MOESM1_ESM.pdf]

Revisiting the functional significance of binocular cues for perceiving motion-in-depth

Supplementary Methods

Detailed Descriptions of the Experiments.

Experiment 1: Full-cue, near vs zero, full- and noise-reference

Fifteen adults (6 male) with an average age of 23.2 years (SD = 4.99) participated. Participants viewed horizontal and vertical displays at 1 meter in which the dots in the test bands underwent in-phase or anti-phase motion. For the horizontal orientation, the anti-phase condition created crossed disparities in the test bands that alternated with zero disparity at 2 Hz. Zero disparity was at the plane of the display. Stimulus displacement in each eye swept from 0.5 to 16 arcmin in 10 equal log steps, which were updated at 1 second intervals. Trials lasted 12 seconds, with the first interval added at both the beginning and end of each trial. This was done to minimize effects of contrast transients when the dots first appeared, and to help ensure that participants did not blink during the middle 10 sec of each trial which went into the data analysis. All displacements are plotted as the single-eye displacement value. The displacement was modulated with a square-wave temporal profile. The reference bands were static in the full-reference conditions and contained interocularly and temporally uncorrelated dots in the noise-reference conditions.

Experiment 2: Full-cue, near vs zero, full- and no-reference

Fifteen adults (8 male, avg. age = 23.4 years, SD = 5.35) participated. No-reference conditions (in which the reference bands were dark empty regions with no dots) replaced noise-reference conditions. All other stimulus features from Experiment 1 were the same.

Experiment 3: Full-cue, near vs far, full- and noise-reference

Fifteen adults (8 male, avg. age=24.2 years, SD=5.81) participated. The end-points of the motion trajectories were set so that for the anti-phase horizontal displacement conditions, the disparity of the test bands alternated between equal crossed and uncrossed values about zero disparity. The magnitude of peak to peak-disparity in the test bands matched that of all other experiments, only the disparity of the test bands relative to that of the reference bands was changed. Noise-reference conditions presented interocularly and temporally uncorrelated dots in the reference bands, as in Experiment 1.

Experiment 4: Full-cue and IOVD-uncorrelated, near vs zero, full-reference

Sixteen adults participated, however one subject was excluded due to their failure to stay awake and engaged during the recording. Fifteen adults (9 male, avg. age = 25.3 years, SD = 5.62) were included in the analysis. The full-cue motion and disparity conditions were the same as in Experiment 1, but the noise-reference conditions were replaced with conditions in which moving test bands contained uncorrelated dots while reference bands contained static dots (IOVD-uncorrelated/full-reference).

Experiment 5: Full-cue and IOVD-anticorrelated, near vs zero, full-reference

Sixteen adults participated, however one subject was excluded from analysis due to the frequency of their eye blinks, which caused more than half of the epochs for every condition across trials to be rejected. Fifteen adults (9 male, avg. age = 27.7 years, SD = 6.31) were analyzed. The full-cue motion and disparity conditions were the same as in Experiment 1, but the absolute motion conditions were replaced with conditions in which moving test bands contained anti-correlated dots while reference bands contained static dots (IOVD-anticorrelated/full-reference). In all conditions, dots were presented on a mean-luminance purple background, which allowed bright and dark dots for both red and blue color channels to be shown to either eye in the anti-correlated display.

Psychophysics Experiment 1: Full-cue, near vs zero, full-reference

Seventeen adults completed this experiment (8 male, avg. age = 26.3 years, SD = 5.48). Viewing distance was set at 3m rather than the 1m used for all other experiments to allow for smaller displacement increments. Spatial frequency of the test and reference bands was adjusted to 0.46 cpd and dot diameter to 4.2 arcmin to match these parameters with those used at 1m. Displacements ranged from 0.16 to 2.56 arcmin in 10 equal log steps. Parameters were otherwise matched to the full-cue/full-reference conditions of Experiments 1 and 2. On each trial, participants viewed either an ascending or descending sweep for a given condition and were instructed to press the right arrow key on a keyboard whenever they detected a state change in the stimuli. For ascending sweeps, participants pressed the key when they first perceived the dots to change from static to moving. For descending sweeps, participants pressed the key when they

perceived the moving dots changed to static. Data from 2 participants were excluded from analysis because they failed to respond or responded incorrectly in more than 15% of trials.

Psychophysics Experiment 2: Full-cue, near vs zero, no-reference

Eighteen adults viewed the second psychophysics experiment, however two subjects were excluded due to the frequency of their blinks during stimulus presentation. Sixteen adults (8 male, avg. age = 24.8 years, SD = 5.59) were analyzed. 8 participants (4 male, avg. age = 28.9, SD = 3.72) took part in both psychophysics experiments. The display parameters matched the full-cue/noise-reference conditions from Experiment 2, except that participants were shown both ascending and descending sweeps. Participants completed the same behavioral measure as described for Psychophysics Experiment 1. We also included infrequent catch trials for ascending and descending conditions, in which the state of motion never changed, i.e. the dots remained static or moving for the duration of the trial. Participants were shown these types of catch trials and understood there should be no response when encountered during the recording session. Catch trials were excluded from the analysis. Data from 1 participant were excluded from analysis because they failed to respond or responded incorrectly in more than 15% of trials.

Infant Experiment: Horizontal full-cue, near vs zero, full- and no-reference

Infants completed a two-part, reduced protocol that consisted of the four horizontal conditions from Experiment 2, but with a displacement range of 2-32 arcmin. The adjusted sweep was used to compensate for the infants' elevated displacement threshold (Norcia, Gerhard and Meredith, 2017). Infants viewed the horizontal full-cue conditions (in-phase and anti-phase) with full-reference and no-reference on separate visits. Condition order was counterbalanced for session one and two. Out of 26 infants, 4 were excluded from the analysis because they were unable to complete at least 5 trials of each condition. Twenty infants (8 male, avg. age = 5.12 months, SD = 1.10) completed both the relative motion and absolute motion session, while another 2 completed only the absolute motion session, for a total of 22 infant datasets (10 male, avg. age =5.47 months, SD = 0.99).

Supplementary Figures

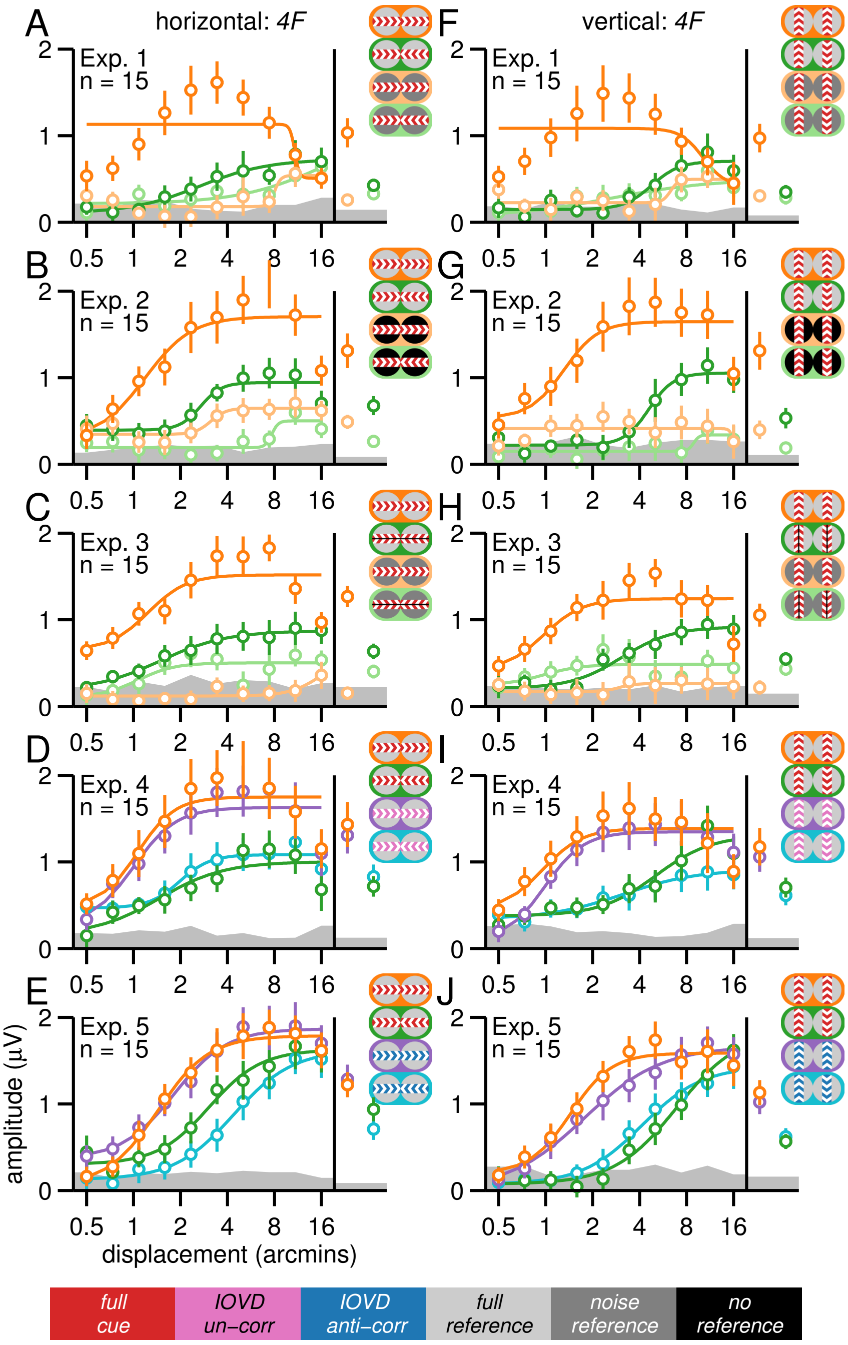

Supplementary Figure 1. Adult fourth harmonic SSVEP response functions. *Panels A-E depict displacement response functions for the horizontal direction of motion and panels F-J the vertical direction of motion. Averages across all displacements are shown on the right side of the response functions. Data are from the first reliable component from an RC analysis derived from 4F data from all conditions, separately for each experiment. Scalp topographies for this component are shown in Supplementary Figure 2. Each experiment had 8 conditions, of which half were horizontal and the rest were vertical. The conditions are represented with icons, with the same color convention as in Figure 2 and the color meanings shown in the legend in the bottom of the figure. Smooth curves are Naka-Rushton function fits to the data and gray bands at the bottom of the plots indicate the average background EEG noise level. Error bars plot +/- 1 standard error of the mean (SEM).*

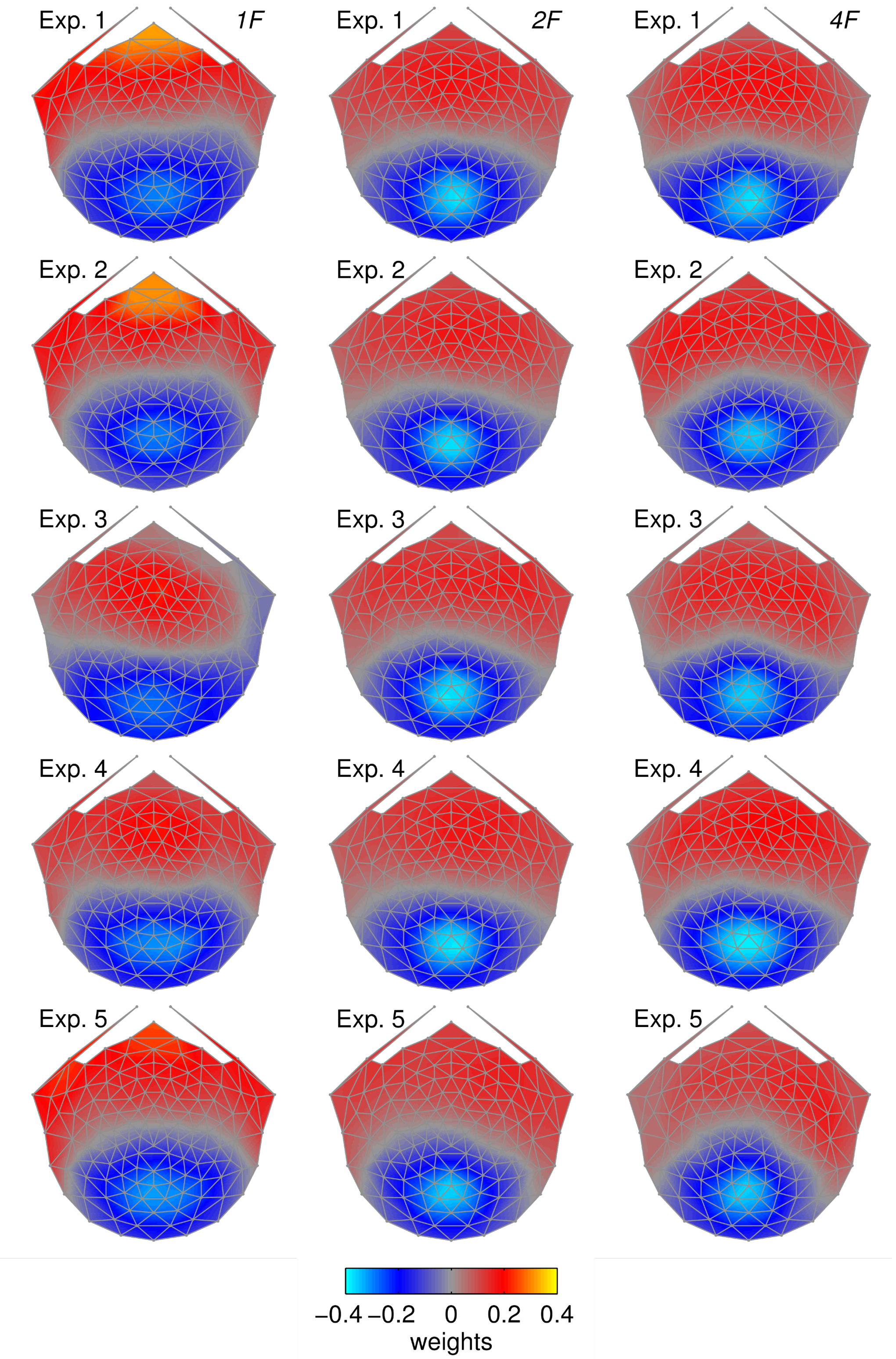

Supplementary Figure 2. Topographies associated with the first reliable component. *The first harmonic (1F) is plotted on the left (data shown in Figure 6), the second harmonic (2F) in the middle (data shown in Figure 2) and the fourth harmonic (4F) on the right (data shown in Supplementary Figure 1). Components were generated by RC analyses run separately for each experiment, and harmonic. The color scale shown in the bottom of the figure indicates the component weights.*

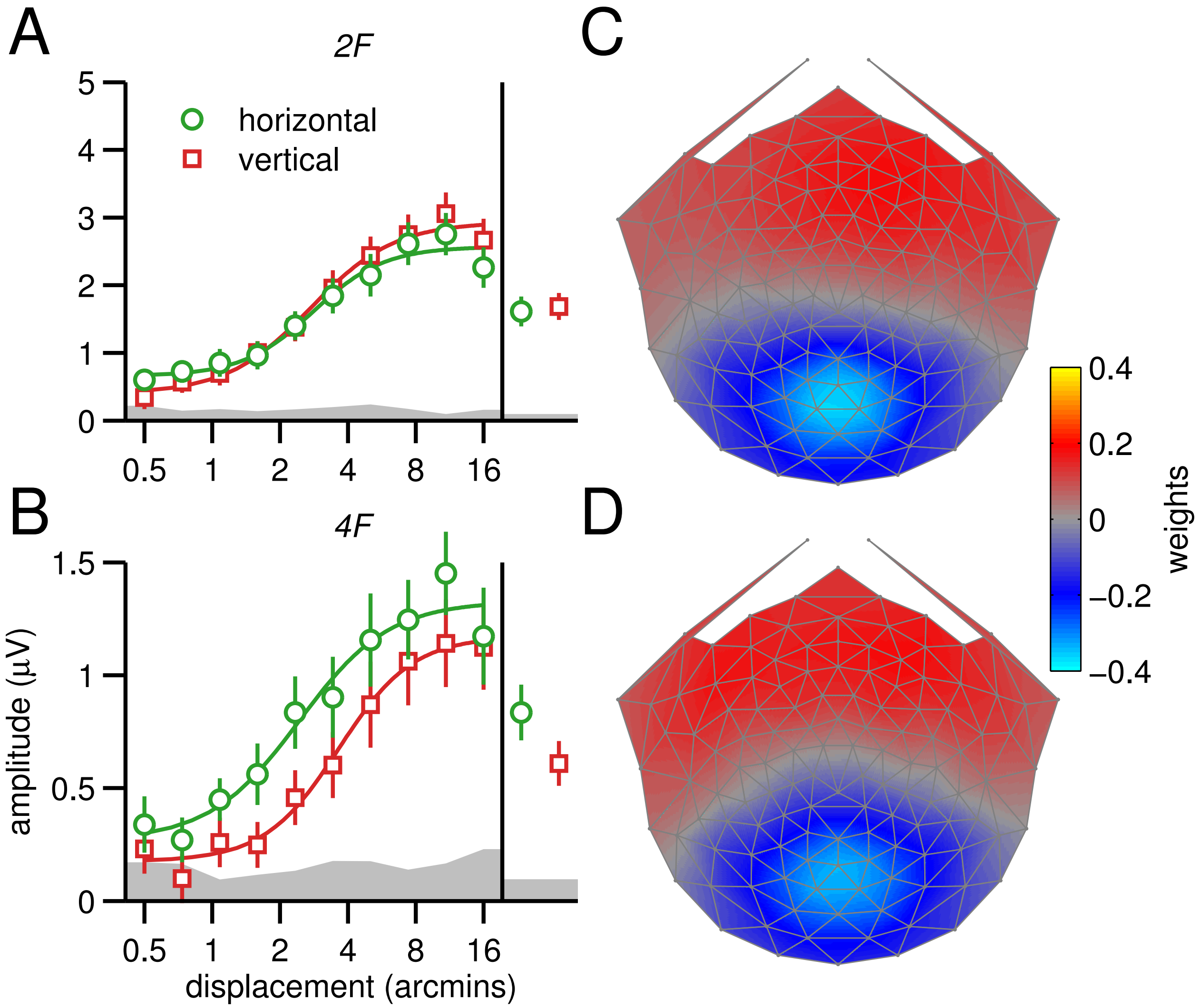

Supplementary Figure 3. Candidate MID signal from IOVD, IOVD isolating conditions. *Response functions for horizontal (green) and vertical (red) anti-phase motion conditions, averaged across the IOVD-isolating conditions of Experiments 4 and 5 (IOVD-u and IOVD-a). The response functions are from the first reliable component of RC analyses done separately on 2F (A) and 4F (B) data, with the topographies shown on the right (C and D). The color scale to the right indicates the values of the weights assigned to each component. The pattern of results is similar to that seen for the full-cue conditions from Experiments 1, 2, 3 and 4 (see Figure 5 in the main paper). Error bars are +/- 1 SEM, smooth curves are Naka-Rushton fits and the gray band indicates the average EEG noise level.*

Supplementary Tables

Horizontal (df=14)	*bin1*	*bin2*	*bin3*	*bin4*	*bin5*	*bin6*	*bin7*	*bin8*	*bin9*	*bin10*	*ave*
disp (arcmins)	0.5	0.73	1.08	1.59	2.33	3.43	5.04	7.41	10.89	16	n/a
Exp. 1 *p*	0.0529	0.003	0.0006	0.0001	<0.0001	<0.0001	<0.0001	0.0001	0.0012	0.0004	<0.0001
Exp. 1 *t*-statistic	2.1145	3.5816	4.412	5.1414	8.994	8.4027	6.9933	5.1978	4.0581	4.564	9.6902
Exp. 1 Cohen's *D*	0.546	0.9248	1.1392	1.3275	2.3222	2.1696	1.8057	1.3421	1.0478	1.1784	2.502
Exp. 2 *p*	0.0592	0.0862	0.0002	<0.0001	<0.0001	<0.0001	<0.0001	<0.0001	<0.0001	<0.0001	<0.0001
Exp. 2 *t*-statistic	2.0532	1.8459	4.9499	9.2555	11.033	9.3816	9.1999	7.6048	8.8969	6.7708	9.7661
Exp. 2 Cohen's *D*	0.5301	0.4766	1.2781	2.3898	2.8487	2.4223	2.3754	1.9636	2.2972	1.7482	2.5216
Exp. 3 *p*	0.0013	<0.0001	<0.0001	<0.0001	<0.0001	<0.0001	<0.0001	<0.0001	<0.0001	<0.0001	<0.0001
Exp. 3 *t*-statistic	3.9993	5.7097	6.7364	5.8581	11.4961	10.2448	11.0267	9.1022	8.8616	8.3264	10.7903
Exp. 3 Cohen's *D*	1.0326	1.4742	1.7393	1.5126	2.9683	2.6452	2.8471	2.3502	2.2881	2.1499	2.7861
Vertical (df=14)	*bin1*	*bin2*	*bin3*	*bin4*	*bin5*	*bin6*	*bin7*	*bin8*	*bin9*	*bin10*	*ave*
disp (arcmins)	0.5	0.73	1.08	1.59	2.33	3.43	5.04	7.41	10.89	16	n/a
Exp. 1 *p*	0.0133	0.0016	0.0001	<0.0001	<0.0001	<0.0001	0.0002	<0.0001	0.0002	0.0002	<0.0001
Exp. 1 *t*-statistic	2.834	3.9098	5.225	7.404	8.4901	8.3752	5.049	5.9479	4.9819	5.0765	8.1899
Exp. 1 Cohen's *D*	0.7317	1.0095	1.3491	1.9117	2.1921	2.1625	1.3036	1.5357	1.2863	1.3107	2.1146
Exp. 2 *p*	0.0791	0.0124	0.0039	0.0003	0.0001	0.0002	<0.0001	<0.0001	<0.0001	0.0002	<0.0001
Exp. 2 *t*-statistic	1.8936	2.8687	3.4463	4.8227	5.3368	4.9335	5.4184	7.4542	6.797	5.0225	6.0057
Exp. 2 Cohen's *D*	0.4889	0.7407	0.8898	1.2452	1.3779	1.2738	1.399	1.9247	1.755	1.2968	1.5507
Exp. 3 *p*	<0.0001	<0.0001	<0.0001	<0.0001	<0.0001	<0.0001	<0.0001	<0.0001	<0.0001	<0.0001	<0.0001
Exp. 3 *t*-statistic	7.5089	6.3871	5.8978	6.25	7.9124	8.5182	7.1912	8.0112	7.9576	7.8629	9.9506
Exp. 3 Cohen's *D*	1.9388	1.6491	1.5228	1.6137	2.043	2.1994	1.8568	2.0685	2.0547	2.0302	2.5692

Supplementary Table 1: Summary of paired two-way *t*-tests comparing full-reference and noise-reference full-cue in-phase motion conditions. Positive *t*-values indicate larger values for full-reference compared to noise-reference. Uncorrected *p*'s < 0.05 are indicated in orange for positive *t*-values and blue for negative *t*-values.

Horizontal (df=14)	*bin1*	*bin2*	*bin3*	*bin4*	*bin5*	*bin6*	*bin7*	*bin8*	*bin9*	*bin10*	*ave*
disp (arcmins)	0.5	0.73	1.08	1.59	2.33	3.43	5.04	7.41	10.89	16	n/a
Exp. 1 *p*	0.0786	0.0487	0.065	0.2091	0.012	0.0005	<0.0001	<0.0001	<0.0001	<0.0001	<0.0001
Exp. 1 *t*-statistic	1.8975	2.1591	2.0025	1.3168	2.885	4.4819	6.0273	5.3716	7.0587	6.0373	6.0382
Exp. 1 Cohen's *D*	0.4899	0.5575	0.5171	0.34	0.7449	1.1572	1.5562	1.3869	1.8226	1.5588	1.5591
Exp. 2 *p*	0.2904	0.0849	0.0031	<0.0001	0.0003	0.0003	<0.0001	<0.0001	0.0023	0.1661	0.0003
Exp. 2 *t*-statistic	1.0987	1.8544	3.5604	5.6053	4.8114	4.7007	5.3953	5.8819	3.7171	1.4608	4.8618
Exp. 2 Cohen's *D*	0.2837	0.4788	0.9193	1.4473	1.2423	1.2137	1.3931	1.5187	0.9597	0.3772	1.2553
Exp. 3 *p*	0.6602	0.8561	0.5802	0.0487	0.0142	0.0003	<0.0001	<0.0001	<0.0001	<0.0001	<0.0001
Exp. 3 *t*-statistic	-0.4491	-0.1848	0.5663	2.159	2.7979	4.803	6.6389	8.4243	7.0222	5.3767	6.2326
Exp. 3 Cohen's *D*	-0.116	-0.0477	0.1462	0.5575	0.7224	1.2401	1.7141	2.1751	1.8131	1.3883	1.6093
Vertical (df=14)	*bin1*	*bin2*	*bin3*	*bin4*	*bin5*	*bin6*	*bin7*	*bin8*	*bin9*	*bin10*	*ave*
disp (arcmins)	0.5	0.73	1.08	1.59	2.33	3.43	5.04	7.41	10.89	16	n/a
Exp. 1 *p*	0.2932	0.163	0.1458	0.4061	0.0017	<0.0001	<0.0001	<0.0001	<0.0001	0.0001	<0.0001
Exp. 1 *t*-statistic	1.0921	1.4725	1.5401	0.8566	3.8574	5.7271	5.5844	5.7849	6.3304	5.1929	5.4918
Exp. 1 Cohen's *D*	0.282	0.3802	0.3976	0.2212	0.996	1.4787	1.4419	1.4937	1.6345	1.3408	1.418
Exp. 2 *p*	0.0704	0.0236	0.0409	0.006	0.0052	0.0386	0.0022	0.0005	0.0036	0.0584	0.0008
Exp. 2 *t*-statistic	1.9583	2.5403	2.2514	3.2326	3.3094	2.2828	3.736	4.4602	3.4969	2.0612	4.2593
Exp. 2 Cohen's *D*	0.5056	0.6559	0.5813	0.8347	0.8545	0.5894	0.9646	1.1516	0.9029	0.5322	1.0998
Exp. 3 *p*	0.8027	0.1397	0.0354	0.0064	0.001	<0.0001	<0.0001	<0.0001	<0.0001	<0.0001	<0.0001
Exp. 3 *t*-statistic	-0.2546	1.5657	2.3287	3.2004	4.1412	5.6363	7.9192	9.1673	9.8211	8.4525	7.2399
Exp. 3 Cohen's *D*	-0.0657	0.4043	0.6013	0.8263	1.0692	1.4553	2.0447	2.367	2.5358	2.1824	1.8693

Supplementary Table 2: Summary of paired two-way *t*-tests comparing full-reference and noise-reference full-cue anti-phase conditions. Positive *t*-values indicate larger values for full-reference compared to noise-reference. Uncorrected *p*'s < 0.05 are indicated in orange for positive *t*-values and blue for negative *t*-values.

Horizontal (df=14)	*bin1*	*bin2*	*bin3*	*bin4*	*bin5*	*bin6*	*bin7*	*bin8*	*bin9*	*bin10*	*ave*
disp (arcmins)	0.5	0.73	1.08	1.59	2.33	3.43	5.04	7.41	10.89	16	n/a
Exp. 1 *p*	0.0996	0.0016	0.0012	0.0016	0.0024	0.0001	0.0137	0.9072	0.088	0.1094	0.0028
Exp. 1 *t*-statistic	1.7635	3.8962	4.056	3.8984	3.6988	5.1723	2.8162	-0.1187	-1.8337	-1.7098	3.6146
Exp. 1 Cohen's *D*	0.4553	1.006	1.0472	1.0066	0.955	1.3355	0.7271	-0.0306	-0.4735	-0.4415	0.9333
Exp. 2 *p*	0.0608	0.0038	<0.0001	<0.0001	0.0004	0.0016	0.001	0.0728	0.4702	0.4487	0.0002
Exp. 2 *t*-statistic	2.0386	3.4686	6.9082	6.156	4.5603	3.8884	4.1602	1.9401	-0.7422	0.7795	4.9374
Exp. 2 Cohen's *D*	0.5264	0.8956	1.7837	1.5895	1.1775	1.004	1.0741	0.5009	-0.1916	0.2013	1.2748
Exp. 3 *p*	0.0002	<0.0001	<0.0001	0.0018	0.0006	0.0022	0.013	0.1467	0.8139	0.9091	0.0008
Exp. 3 *t*-statistic	5.0041	7.3395	7.4514	3.8316	4.43	3.7445	2.8422	1.5364	-0.2399	0.1163	4.251
Exp. 3 Cohen's *D*	1.292	1.8951	1.9239	0.9893	1.1438	0.9668	0.7338	0.3967	-0.0619	0.03	1.0976
Exp. 4 *p*	0.0254	0.0148	0.0008	0.0003	0.0009	0.0048	0.0019	0.016	0.162	0.8928	0.0017
Exp. 4 *t*-statistic	2.5011	2.7783	4.2568	4.6991	4.1913	3.3446	3.8171	2.7401	1.4762	0.1373	3.8769
Exp. 4 Cohen's *D*	0.6458	0.7173	1.0991	1.2133	1.0822	0.8636	0.9856	0.7075	0.3811	0.0354	1.001
Exp. 5 *p*	0.9477	0.2118	0.0049	0.0244	0.0104	0.0527	0.577	0.7563	0.3509	0.252	0.2541
Exp. 5 *t*-statistic	-0.0668	1.3083	3.3327	2.5231	2.9583	2.1167	0.5711	-0.3165	-0.965	-1.1948	1.1892
Exp. 5 Cohen's *D*	-0.0172	0.3378	0.8605	0.6515	0.7638	0.5465	0.1475	-0.0817	-0.2492	-0.3085	0.3071
Vertical (df=14)	*bin1*	*bin2*	*bin3*	*bin4*	*bin5*	*bin6*	*bin7*	*bin8*	*bin9*	*bin10*	*ave*
disp (arcmins)	0.5	0.73	1.08	1.59	2.33	3.43	5.04	7.41	10.89	16	n/a
Exp. 1 *p*	0.0003	0.0009	0.0002	0.0001	0.0005	0.0004	0.0138	0.9432	0.3744	0.1682	<0.0001
Exp. 1 *t*-statistic	4.6885	4.2115	4.9299	5.1858	4.4994	4.6493	2.8141	-0.0725	-0.9175	-1.4532	5.4548
Exp. 1 Cohen's *D*	1.2106	1.0874	1.2729	1.339	1.1618	1.2005	0.7266	-0.0187	-0.2369	-0.3752	1.4084
Exp. 2 *p*	0.131	0.0271	0.0033	0.0015	0.0006	0.0077	0.0373	0.0732	0.595	0.6747	0.0114
Exp. 2 *t*-statistic	1.6042	2.4675	3.5302	3.9187	4.434	3.1072	2.3002	1.937	0.544	-0.4286	2.9126
Exp. 2 Cohen's *D*	0.4142	0.6371	0.9115	1.0118	1.1448	0.8023	0.5939	0.5001	0.1405	-0.1107	0.752
Exp. 3 *p*	0.0006	0.0002	0.0031	0.018	0.1491	0.4229	0.3247	0.1822	0.4084	0.1152	0.0444
Exp. 3 *t*-statistic	4.405	4.9883	3.5697	2.6794	1.5266	0.8256	-1.0207	-1.4037	-0.8524	-1.6796	2.2088
Exp. 3 Cohen's *D*	1.1374	1.288	0.9217	0.6918	0.3942	0.2132	-0.2635	-0.3624	-0.2201	-0.4337	0.5703
Exp. 4 *p*	0.0043	0.0074	0.001	0.0015	0.0001	0.0023	0.0355	0.184	0.4842	0.1818	0.0026
Exp. 4 *t*-statistic	3.3973	3.1273	4.1258	3.9382	5.2651	3.7085	2.3269	1.3977	0.7185	1.4051	3.6605
Exp. 4 Cohen's *D*	0.8772	0.8075	1.0653	1.0168	1.3594	0.9575	0.6008	0.3609	0.1855	0.3628	0.9451
Exp. 5 *p*	0.4197	0.0069	0.0022	0.0001	<0.0001	<0.0001	0.0028	0.2301	0.7228	0.4277	0.0005
Exp. 5 *t*-statistic	0.8315	3.1609	3.7485	5.2235	7.7072	5.5533	3.6187	1.2546	0.3619	0.8168	4.4561
Exp. 5 Cohen's *D*	0.2147	0.8161	0.9678	1.3487	1.99	1.4338	0.9343	0.3239	0.0934	0.2109	1.1506

Supplementary Table 3: Summary of paired two-way *t*-tests comparing full-cue/full-reference in-phase and anti-phase motion conditions. Positive *t*-values indicate larger values for in-phase compared to anti-phase motion. Uncorrected *p*'s < 0.05 are indicated in orange for positive *t*-values and blue for negative *t*-values.

Horizontal (df=14)	*bin1*	*bin2*	*bin3*	*bin4*	*bin5*	*bin6*	*bin7*	*bin8*	*bin9*	*bin10*	*ave*
disp (arcmins)	0.5	0.73	1.08	1.59	2.33	3.43	5.04	7.41	10.89	16	n/a
Exp. 2 *p*	0.0914	0.0193	0.0028	0.1907	0.6093	0.3693	0.0324	0.0007	0.0012	0.0027	0.1717
Exp. 2 *t*-statistic	1.8124	2.6434	3.6146	1.3751	0.5228	-0.9277	-2.375	-4.3354	-4.0498	-3.6368	-1.4406
Exp. 2 Cohen's *D*	0.4679	0.6825	0.9333	0.3551	0.135	-0.2395	-0.6132	-1.1194	-1.0456	-0.939	-0.372
Vertical (df=14)	*bin1*	*bin2*	*bin3*	*bin4*	*bin5*	*bin6*	*bin7*	*bin8*	*bin9*	*bin10*	*ave*
disp (arcmins)	0.5	0.73	1.08	1.59	2.33	3.43	5.04	7.41	10.89	16	n/a
Exp. 2 *p*	0.0187	0.0408	0.072	0.1836	0.1946	0.0128	0.0003	<0.0001	<0.0001	0.0108	0.0003
Exp. 2 *t*-statistic	2.658	2.2537	1.9458	1.3988	-1.3623	-2.8514	-4.7103	-5.6349	-7.5685	-2.936	-4.7474
Exp. 2 Cohen's *D*	0.6863	0.5819	0.5024	0.3612	-0.3517	-0.7362	-1.2162	-1.4549	-1.9542	-0.7581	-1.2258

Supplementary Table 4: Statistical results of paired two-way *t*-test comparing full-cue/no-reference in-phase and anti-phase motion. Positive *t*-values indicate larger values for in-phase compared to anti-phase motion. Uncorrected *p*'s < 0.05 are indicated in orange for positive *t*-values and blue for negative *t*-values. Note that both horizontal and vertical conditions produce negative *t*-values, indicating larger responses to anti-phase compared to in-phase.

Horizontal (df=14)	*bin1*	*bin2*	*bin3*	*bin4*	*bin5*	*bin6*	*bin7*	*bin8*	*bin9*	*bin10*	*ave*
disp (arcmins)	0.5	0.73	1.08	1.59	2.33	3.43	5.04	7.41	10.89	16	n/a
IOVD-u *p*	0.5083	0.0528	0.0085	0.0019	0.0094	0.027	0.0017	0.0123	0.0513	0.3131	0.0039
IOVD-u *t*-statistic	0.6788	2.1152	3.0588	3.8168	3.0055	2.47	3.8564	2.8739	2.1311	1.0495	3.4556
IOVD-u Cohen's *D*	0.1753	0.5462	0.7898	0.9855	0.776	0.6378	0.9957	0.742	0.5503	0.2805	0.8922
IOVD-a *p*	0.0843	0.0269	0.01	0.0318	0.1794	0.1245	0.1324	0.0304	0.1689	0.1732	0.0214
IOVD-a *t*-statistic	1.8579	2.4717	2.9767	2.3853	1.4134	1.6341	1.5977	2.408	1.4507	1.4353	2.5891
IOVD-a Cohen's *D*	0.4797	0.6382	0.7686	0.6159	0.3649	0.4219	0.4125	0.6218	0.3746	0.3706	0.6685
Vertical (df=14)	*bin1*	*bin2*	*bin3*	*bin4*	*bin5*	*bin6*	*bin7*	*bin8*	*bin9*	*bin10*	*ave*
disp (arcmins)	0.5	0.73	1.08	1.59	2.33	3.43	5.04	7.41	10.89	16	n/a
IOVD-u *p*	0.0877	0.3514	0.0122	0.0006	0.0006	0.0742	0.045	0.1332	0.3888	0.4073	0.0076
IOVD-u *t*-statistic	1.8359	0.964	2.8751	4.3691	4.4219	1.9291	2.2008	1.5941	0.8895	0.8544	3.1179
IOVD-u Cohen's *D*	0.474	0.2489	0.7424	1.1281	1.1417	0.4981	0.5682	0.4116	0.2297	0.2206	0.805
IOVD-a *p*	0.7915	0.0677	0.0572	0.0003	0.0004	0.027	0.023	0.0032	0.0668	0.0144	0.0024
IOVD-a *t*-statistic	0.2694	1.9803	2.072	4.8034	4.6303	2.4689	2.5535	3.5488	1.9871	2.7937	3.7044
IOVD-a Cohen's *D*	0.0696	0.5113	0.535	1.2402	1.1955	0.6375	0.6593	0.9163	0.5131	0.7213	0.9565

Supplementary Table 5: Summary of paired two-way *t*-tests comparing IOVD-isolating in-phase and anti-phase conditions. Data from two sets of conditions: IOVD-uncorrelated (IOVD-u, run in Experiment 4) and IOVD-anticorrelated (IOVD-a, run in Experiment 5). Positive *t*-values indicate larger values for in-phase compared to anti-phase motion. Uncorrected *p*'s < 0.05 are indicated in orange for positive *t*-values and blue for negative *t*-values.

EEG recorded during psychophysics: full-cue/full-reference											
Horizontal (df=14)	*bin1*	*bin2*	*bin3*	*bin4*	*bin5*	*bin6*	*bin7*	*bin8*	*bin9*	*bin10*	*ave*
disp (arcmins)	0.16	0.22	0.3	0.4	0.55	0.75	1.02	1.38	1.88	2.56	n/a
p	0.2559	0.8366	0.9064	0.3328	0.001	0.0004	<0.0001	<0.0001	<0.0001	0.0005	<0.0001
t-statistic	1.1845	0.2101	0.1198	1.0031	4.1636	4.6651	5.4558	6.1189	5.6719	4.5386	5.7912
Cohen's *D*	0.3058	0.0543	0.0309	0.259	1.075	1.2045	1.4087	1.5799	1.4645	1.1719	1.4953
Vertical (df=14)	*bin1*	*bin2*	*bin3*	*bin4*	*bin5*	*bin6*	*bin7*	*bin8*	*bin9*	*bin10*	*ave*
disp (arcmins)	0.16	0.22	0.3	0.4	0.55	0.75	1.02	1.38	1.88	2.56	n/a
p	0.6376	0.7829	0.2551	0.221	0.0152	0.0007	<0.0001	<0.0001	<0.0001	<0.0001	<0.0001
t-statistic	0.4816	0.281	1.1866	1.2811	2.7636	4.3569	7.5686	6.9216	5.7555	6.9508	6.4205
Cohen's *D*	0.1243	0.0725	0.3064	0.3308	0.7135	1.125	1.9542	1.7872	1.4861	1.7947	1.6578
EEG recorded during psychophysics: full-cue/no-reference											
Horizontal (df=14)	*bin1*	*bin2*	*bin3*	*bin4*	*bin5*	*bin6*	*bin7*	*bin8*	*bin9*	*bin10*	*ave*
disp (arcmins)	0.5	0.73	1.08	1.59	2.33	3.43	5.04	7.41	10.89	16	n/a
p	0.0852	0.1738	0.2821	0.2123	0.0557	0.0291	0.016	0.0282	0.0054	0.0044	0.0075
t-statistic	1.8524	1.4331	1.1187	-1.307	-2.0866	-2.4301	-2.7391	-2.4473	-3.285	-3.3885	-3.1185
Cohen's *D*	0.4783	0.37	0.2888	-0.3375	-0.5388	-0.6275	-0.7072	-0.6319	-0.8482	-0.8749	-0.8052
Vertical (df=14)	*bin1*	*bin2*	*bin3*	*bin4*	*bin5*	*bin6*	*bin7*	*bin8*	*bin9*	*bin10*	*ave*
disp (arcmins)	0.5	0.73	1.08	1.59	2.33	3.43	5.04	7.41	10.89	16	n/a
p	0.9264	0.9524	0.7738	0.6227	0.0546	0.0345	0.0042	0.0023	0.0054	0.0015	0.0169
t-statistic	-0.0941	-0.0608	0.293	-0.5031	-2.0972	-2.3412	-3.4134	-3.7202	-3.284	-3.9395	-2.7113
Cohen's *D*	-0.0243	-0.0157	0.0757	-0.1299	-0.5415	-0.6045	-0.8813	-0.9606	-0.8479	-1.0172	-0.7

Supplementary Table 6: Results of paired two-way *t*-tests done on the *2F* data recorded during the two psychophysical experiments, comparing in-phase and anti-phase full-cue conditions with full-reference and no-reference. Positive *t*-values indicate larger values for in-phase compared to anti-phase motion. Uncorrected p's < 0.05 are indicated in orange for positive *t*-values and blue for negative *t*-values. Note that the no-reference conditions produce negative *t*-values indicating larger responses to anti-phase compared to in-phase.

in-phase full-cue: full-reference vs no-reference											
df=19	*bin1*	*bin2*	*bin3*	*bin4*	*bin5*	*bin6*	*bin7*	*bin8*	*bin9*	*bin10*	*ave*
disp (arcmins)	2	2.72	3.7	5.04	6.86	9.33	12.7	17.28	23.52	32	n/a
p	0.8671	0.0778	0.0135	0.1258	0.7232	0.6733	0.205	0.935	0.7043	0.8348	0.262
t-statistic	0.1696	-1.8645	-2.7236	-1.6016	0.3594	-0.4282	-1.3125	0.0826	-0.3852	-0.2114	-1.1559
Cohen's *D*	0.0379	-0.4169	-0.609	-0.3581	0.0804	-0.0957	-0.2935	0.0185	-0.0861	-0.0473	-0.2585
anti-phase full-cue: full-reference vs no-reference											
df=19	*bin1*	*bin2*	*bin3*	*bin4*	*bin5*	*bin6*	*bin7*	*bin8*	*bin9*	*bin10*	*ave*
disp (arcmins)	2	2.72	3.7	5.04	6.86	9.33	12.7	17.28	23.52	32	n/a
p	0.3999	0.8149	0.1703	0.3298	0.9718	0.8854	0.4425	0.577	0.9683	0.9256	0.8257
t-statistic	-0.8611	-0.2374	-1.4254	-1.0001	-0.0358	-0.1461	0.7843	0.5675	0.0403	-0.0946	-0.2233
Cohen's *D*	-0.1925	-0.0531	-0.3187	-0.2236	-0.008	-0.0327	0.1754	0.1269	0.009	-0.0212	-0.0499
full-cue/full-reference: in-phase vs anti-phase											
df=19	*bin1*	*bin2*	*bin3*	*bin4*	*bin5*	*bin6*	*bin7*	*bin8*	*bin9*	*bin10*	*ave*
disp (arcmins)	2	2.72	3.7	5.04	6.86	9.33	12.7	17.28	23.52	32	n/a
p	0.0075	<0.0001	<0.0001	<0.0001	0.0013	0.0009	<0.0001	0.0025	0.0258	0.0168	<0.0001
t-statistic	-2.9932	-5.1199	-4.9471	-4.9882	-3.7534	-3.9422	-5.4804	-3.4895	-2.4185	-2.6206	-6.3739
Cohen's *D*	-0.6693	-1.1448	-1.1062	-1.1154	-0.8393	-0.8815	-1.2255	-0.7803	-0.5408	-0.586	-1.4253
full-cue/no-reference: in-phase vs anti-phase											
df=21	*bin1*	*bin2*	*bin3*	*bin4*	*bin5*	*bin6*	*bin7*	*bin8*	*bin9*	*bin10*	*ave*
disp (arcmins)	2	2.72	3.7	5.04	6.86	9.33	12.7	17.28	23.52	32	n/a
p	0.0029	0.0024	0.0001	0.0034	0.0017	0.003	0.0139	0.0112	0.0464	0.0256	<0.0001
t-statistic	-3.3682	-3.4459	-4.7767	-3.2975	-3.5904	-3.3501	-2.685	-2.7807	-2.1171	-2.4022	-5.4511
Cohen's *D*	-0.7181	-0.7347	-1.0184	-0.703	-0.7655	-0.7142	-0.5724	-0.5928	-0.4514	-0.5122	-1.1622

Supplementary Table 7: Results of paired two-way *t*-tests done on the *2F* data from the infants. Four distinct comparisons were done. For the comparisons of full-reference and no-reference conditions (top half), positive *t*-values indicate larger values for full-reference compared to no-reference, for the comparisons of in-phase and anti-phase (bottom half), positive *t*-values indicate larger values for in-phase compared to anti-phase motion. Uncorrected *p*'s < 0.05 are indicated in orange for positive *t*-values and blue for negative *t*-values. Note that both full-reference and no-reference conditions produce negative *t*-values indicating larger responses to anti-phase compared to in-phase.

Exp. 1-4 (full-cue/full-reference) df=41	*bin1*	*bin2*	*bin3*	*bin4*	*bin5*	*bin6*	*bin7*	*bin8*	*bin9*	*bin10*	*ave*
disp (arcmins)	0.5	0.73	1.08	1.59	2.33	3.43	5.04	7.41	10.89	16	n/a
2F p	0.0276	0.7386	0.6616	0.6878	0.7777	0.4615	0.1619	0.6983	0.0553	0.5889	0.7431
2F t-value	2.2839	0.3359	-0.4409	0.4047	-0.2842	-0.7434	-1.4243	0.3904	1.9727	0.5448	0.33
2F Cohen's D	0.3524	0.0518	-0.068	0.0625	-0.0439	-0.1147	-0.2198	0.0602	0.3044	0.0841	0.0509
4F p	0.3896	0.1129	0.0083	0.0139	0.0034	0.0192	0.0246	0.752	0.2505	0.1393	0.0789
4F t-value	-0.8695	1.62	2.7757	2.5693	3.1127	2.4384	2.3338	0.3181	-1.1656	-1.5078	1.8021
4F Cohen's D	-0.1342	0.25	0.4283	0.3965	0.4803	0.3763	0.3601	0.0491	-0.1799	-0.2327	0.2781
Exp. 4-5 (IOVD/full-reference) df=23	*bin1*	*bin2*	*bin3*	*bin4*	*bin5*	*bin6*	*bin7*	*bin8*	*bin9*	*bin10*	*ave*
disp (arcmins)	0.5	0.73	1.08	1.59	2.33	3.43	5.04	7.41	10.89	16	n/a
2F p	0.1044	0.3236	0.2408	0.7919	0.8866	0.5535	0.1119	0.4371	0.0606	0.0052	0.4808
2F t-value	1.6907	1.0088	1.204	-0.2669	0.1441	-0.6013	-1.6533	-0.7909	-1.9732	-3.1013	-0.7166
2F Cohen's D	0.3451	0.2059	0.2458	-0.0545	0.0294	-0.1227	-0.3375	-0.1615	-0.4028	-0.6467	-0.1463
4F p	0.3787	0.1502	0.1761	0.0319	0.0046	0.0376	0.0983	0.2857	0.031	0.9386	0.0053
4F t-value	0.8976	1.4884	1.3957	2.2844	3.1427	2.2059	1.7231	1.0931	2.2981	-0.0779	3.0768
4F Cohen's D	0.1832	0.3038	0.2849	0.4663	0.6415	0.4503	0.3517	0.2231	0.4691	-0.0162	0.628

Supplementary Table 8: Results of paired two-way *t*-tests comparing horizontal and vertical referenced anti-phase conditions, for the second (*2F*) and fourth (*4F*) harmonic. The comparison was made separately on the full-cue conditions from Experiments 1-4 (top half) and the IOVD-isolating conditions of Experiments 4 and 5 (bottom half). Positive *t*-values indicate larger values for horizontal compared to vertical motion. Uncorrected *p*'s < 0.05 are indicated in orange for positive *t*-values and blue for negative *t*-values.
