# Supplementary material for: Revisiting the functional significance of binocular cues for perceiving motion-in-depth
Source: Nat Commun. 2018 Aug 29;9:3511. doi: 10.1038/s41467-018-05918-7 (PMC6115357; doi:10.1038/s41467-018-05918-7)
Supplement: Supplementary file 3 — Description of Additional Supplementary Files [file 41467_2018_5918_MOESM3_ESM.pdf]

Description of Additional Supplementary Files

File Name: Supplementary Movie 1
Description: A single trial of the horizontal full cue/full reference in-phase motion condition run in Experiments 1, 2, 3, and 4. Note that the first and last second of the trial, where all dots were static, were excluded from the analysis.

File Name: Supplementary Movie 2
Description: A single trial of the horizontal full cue/full reference anti-phase motion condition run in Experiments 1, 2, and 4. Note that the first and last second of the trial, where all dots were static, were excluded from the analysis. Red/blue anaglyph glasses are required to view the disparities.

File Name: Supplementary Movie 3
Description: A single trial of the vertical full cue/full reference in-phase motion condition run in Experiments 1, 2, 3, and 4. Note that the first and last second of the trial, where all dots were static, were excluded from the analysis.

File Name: Supplementary Movie 4
Description: A single trial of the vertical full cue/full reference anti-phase motion condition run in Experiments 1, 2, and 4. Note that the first and last second of the trial, where all dots were static, were excluded from the analysis. Red/blue anaglyph glasses are required to view the disparities.
